# Highly Plasmonic Titanium Nitride by Room-Temperature Sputtering

**DOI:** 10.1038/s41598-019-51236-3

**Published:** 2019-10-25

**Authors:** Chun-Chieh Chang, John Nogan, Zu-Po Yang, Wilton J. M. Kort-Kamp, Willard Ross, Ting S. Luk, Diego A. R. Dalvit, Abul K. Azad, Hou-Tong Chen

**Affiliations:** 1 Center for Integrated Nanotechnologies, Los Alamos National Laboratory, Los Alamos, New Mexico 87545 USA; 2 Institute of Electro-Optical Engineering, National Taiwan Normal University, Taipei, 11677 Taiwan; 3 Center for Integrated Nanotechnologies, Sandia National Laboratories, Albuquerque, New Mexico 87123 USA; 4 Institute of Photonic System, National Chiao Tung University, 301 Gaofa 3rd. Road, Tainan, 71150 Taiwan; 5 Theoretical Division, Los Alamos National Laboratory, Los Alamos, New Mexico 87545 USA; 6 Center for Nonlinear Studies, Los Alamos National Laboratory, Los Alamos, New Mexico 87545 USA

**Keywords:** Optical materials and structures, Nanophotonics and plasmonics

## Abstract

Titanium nitride (TiN) has recently emerged as an attractive alternative material for plasmonics. However, the typical high-temperature deposition of plasmonic TiN using either sputtering or atomic layer deposition has greatly limited its potential applications and prevented its integration into existing CMOS device architectures. Here, we demonstrate highly plasmonic TiN thin films and nanostructures by a room-temperature, low-power, and bias-free reactive sputtering process. We investigate the optical properties of the TiN films and their dependence on the sputtering conditions and substrate materials. We find that our TiN possesses one of the largest negative values of the real part of the dielectric function as compared to all other plasmonic TiN films reported to date. Two-dimensional periodic arrays of TiN nanodisks are then fabricated, from which we validate that strong plasmonic resonances are supported. Our room-temperature deposition process can allow for fabricating complex plasmonic TiN nanostructures and be integrated into the fabrication of existing CMOS-based photonic devices to enhance their performance and functionalities.

## Introduction

Titanium nitride (TiN)^1^, along with other transition metal nitrides, has recently emerged as promising alternative plasmonic materials at visible and near-infrared wavelengths^2–4^. Although its loss is yet to be comparable to conventional plasmonic materials represented by noble metals (gold and silver), TiN possesses tunable optical properties, and is cost effective, chemically and thermally stable, and compatible with modern silicon fabrication technologies. Recent studies have shown that TiN can enable plasmonic performance comparable to that of gold, and is a better material candidate for hyperbolic metamaterials, transformation optics, and photothermal energy conversion^2,5–7^. To date, plasmonic TiN has been employed in a wide variety of applications including broadband absorbers^8^, spectrally selective infrared thermal emitters^9^, hyperbolic metamaterials^5,10^, local heating sources^11,12^, self-powered plasmonic photodetectors^13,14^, and nonlinear optics^15^.

Low-temperature growth of TiN is highly desirable to make it compatible with the complementary metal-oxide-semiconductor (CMOS) fabrication. Low-temperature plasmonic TiN deposition has been recently reported by optimizing the plasma exposure per growth cycle during plasma-enhanced atomic layer deposition (PEALD) at 250 °C^16^; however, most of the TiN film deposition has been adopting a high-temperature sputtering process^2^ to achieve the plasmonic character (i.e., negative real part of the dielectric function, *ε*_1_, at wavelengths larger than the screened plasma wavelength, *λ*_ps_). Sputtering process represents a mature and low-cost deposition approach, but the high-temperature has limited potential applications of plasmonic TiN, and prevented its integration into existing CMOS device architectures. There has been much effort in enabling room-temperature deposition of plasmonic TiN films. Edlou *et al*.^17^ reported over two decades ago the preparation of plasmonic TiN thin films by a DC magnetron reactive sputtering process, without intentionally heating or biasing the substrate during deposition. However, their effort was mainly aimed at the development of TiN films with desirable properties for harsh environments (e.g., mechanical abrasion and thermal annealing) by varying deposition conditions. It was later shown that significantly increasing the sputtering power (7 kW)^18^ can result in golden-colored plasmonic TiN films, which can also be realized by applying a high impulse sputtering power (>4 MW)^19^ or a substrate bias voltage^20^, yet at the expense of lower crystalline order, increased surface roughness and more defects. Recently, Wang *et al*.^21^ also reported the realization of plasmonic TiN films by DC magnetron reactive sputtering at room temperature. Yet, without annealing at 700 °C or higher temperatures, their plasmonic TiN films, instead of having gold-like luster, appear to be red. The measured *λ*_ps_ of the as-deposited TiN films, regardless of film thickness, is beyond 700 nm, indicating that they are unsuitable for plasmonic applications in the visible region.

Here, we report on the realization of plasmonic TiN thin films by a room-temperature, low-power, and bias-free reactive sputtering process. We show that highly plasmonic TiN films, i.e., TiN films with large negative real part of the dielectric function *ε*_1_ at near- and mid-infrared wavelengths, can be realized on various substrate surfaces, and their plasmonic characteristics can be tailored through varying the reactive gas flow ratio. We find that our TiN possesses one of the largest negative values of *ε*_1_ as compared to all other plasmonic TiN films reported to date. We further fabricate two-dimensional periodic square arrays of TiN nanodisks, and validate from transmittance measurements that they support strong plasmonic resonances in the visible and near-infrared regions. The room-temperature sputtering process of TiN could be employed to ease the fabrication of more complex TiN-based nanostructures and potentially be integrated into modern CMOS processes to expand the practical applications of plasmonics.

## Results and Discussion

**Figure 1:**
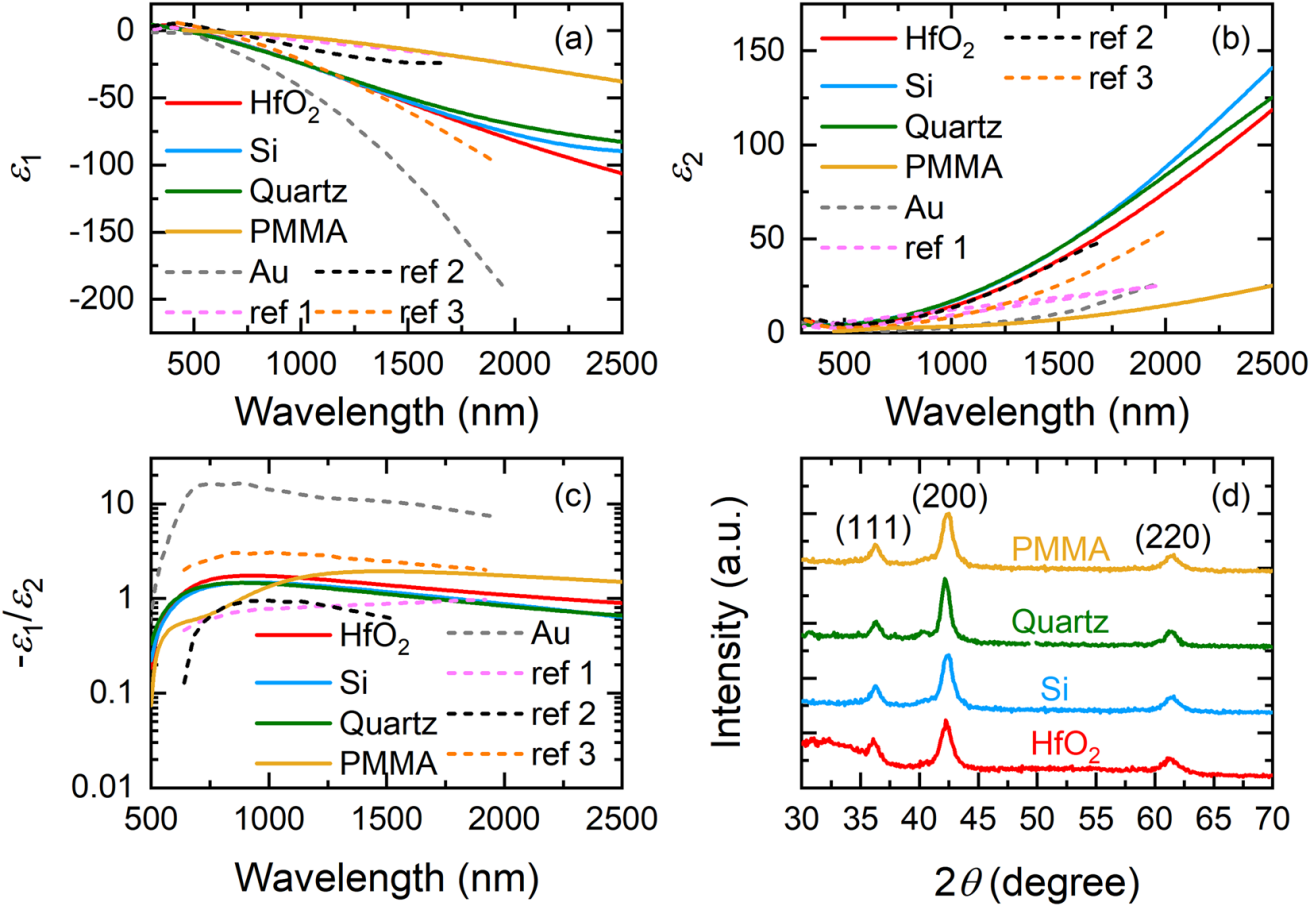
(**a**) Real part *ε*_1_ and (**b**) imaginary part *ε*_2_ of extracted complex dielectric functions of room-temperature sputtered TiN films on different substrate surfaces. Dashed lines in (**a**) and (**b**): *ε*_1_ and *ε*_2_ of TiN (ref 1^3^, ref 2^16^, and ref 3^5^) and Au films from literatures. (**c**) Figure of merit (FOM) − *ε*_1_/*ε*_2_ of the corresponding sputtered TiN films. Dashed lines in (**c**): FOM of TiN and Au films from literatures. (**d**) X-ray diffraction spectra of the corresponding sputtered TiN films. All Spectra are vertically offset for clarity.

**Figure 2:**
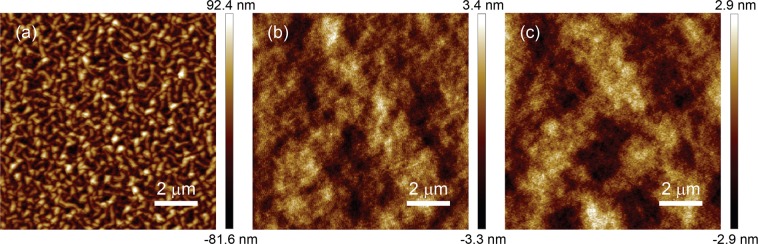
AFM images of Ar:N_2_ = 80%:20% TiN films on different substrate surfaces: (**a**) PMMA, (**b**) HfO_2_, and (**c**) Si.

Figure 1(a,b) show respectively the real (*ε*_1_) and imaginary (*ε*_2_) part of the extracted dielectric functions of TiN films (thickness ~50 nm) deposited on various substrate surfaces at Ar:N_2_ = 80%:20% (except for the TiN films on quartz deposited at Ar:N_2_ = 95%:5%) using Drude-Lorentz model. All TiN films are clearly plasmonic in the spectral region of interest. In particular, the large negative values of *ε*_1_ for TiN deposited on hafnium dioxide (HfO_2_), silicon (Si), and quartz indicate that these films are highly plasmonic. Both of their *ε*_1_ and *ε*_2_ exhibit similar characteristics, and the variation of their *λ*_ps_ is less than 20 nm (*λ*_ps_ = 480, 470, and 460 nm for TiN deposited on HfO_2_, Si, and quartz, respectively), showing that our room-temperature sputtered TiN films have a weak dependence for these substrate surfaces as those prepared by ALD^22^. Note that *λ*_ps_ of these films occurs at wavelengths shorter than that of the ALD plasmonic TiN^16,22^, therefore enabling a broader plasmonic response in the visible region. TiN films on poly(methyl methacrylate) (PMMA) possess a quite distinct dielectric function from others and are less plasmonic, which may have been caused by the surface roughness of the PMMA films induced by the ion bombardment during the sputtering process. As can be seen in the atomic force microscopy (AFM) images (see Fig. 2), the TiN films deposited on PMMA are fairly rough, possessing a root-mean-square roughness value of 26.4 nm, much greater than that for the films deposited on HfO_2_ (0.953 nm) and Si (0.888 nm) surfaces. It is expected that the optical properties of TiN films on PMMA are different since their roughness is essentially on the same order of the film thickness. Nevertheless, our results show that the room-temperature sputtering process can be employed to fabricate plasmonic TiN on unconventional polymeric or elastomeric substrate for flexible plasmonics and nanophotonics applications^23^, which is not possible by either high-temperature sputtering^2,3^ or low-temperature ALD^16^.

We plot for comparison in Fig. 1(a,b) the *ε*_1_ and *ε*_2_ of TiN films deposited by high-temperature sputtering, low-temperature ALD, and high-temperature epitaxial growth, in dashed lines denoted as ref 1^3^, ref 2^16^, and ref 3^5^, respectively. Epitaxial TiN films grown on lattice-matched substrate to our knowledge possess the largest negative value of *ε*_1_ among all plasmonic TiN films reported so far due to their high crystalline quality and low roughness, although they need to be grown at a fairly high temperature (750 °C). Our room-temperature sputtered TiN films are apparently comparable to the epitaxial TiN and superior to films prepared by both high-temperature sputtering and low-temperature ALD in plasmonic character. We believe that the strong plasmonic character of our room-temperature sputtered TiN films is enabled by the close proximity of our samples to the plasma during deposition providing the necessary high energy reactive ions and activated species in lieu of substrate heating or biasing^24^. Note that the observed strong plasmonic character of our TiN is in sharp contrast to the dielectric properties of titanium oxynitride (TiO_x_N_y_)^25^, which usually exhibits double epsilon-near-zero (ENZ) characteristics without substrate heating and special chamber preparation. We also plot in the same figures the optical data of gold (Au) reported by Johnson and Christy^26^. Despite its several disadvantages for plasmonic applications^4^, Au still has better plasmonic character (more negative *ε*_1_) and a smaller loss (smaller *ε*_2_) compared to TiN, which can be further verified by examining the general figure of merits (FOMs) for localized surface plasmonic resonance (LSPR) defined as − *ε*_1_/*ε*_2_^27^, as shown in Fig. 1(c). However, for plasmonic induced hot-carrier generation in photothermal or photoelectric applications^28,29^ where plasmons are resonantly excited by incident light and subsequently decay nonradiatively into hot carriers on an ultrafast time scale, it is desirable to have plasmonic materials possessing not only a large negative *ε*_1_ but also a large *ε*_2_ value. Our TiN films are clearly most suitable for hot-carrier applications in comparison with Au and all the other TiN films, allowing for both strong plasmonic coupling (due to larger negative *ε*_1_) and fast plasmon decay (due to larger positive *ε*_2_). In Fig. 1(d) we show the x-ray diffraction (XRD) spectra of TiN films sputtered on different substrate surfaces. All TiN films possess essentially the same crystalline structure, exhibiting a predominant peak at ~42.5° along with two weak peaks at ~36.2° and ~61.6°, corresponding to the (200), (111), and (220) orientation, respectively. The observation of these peaks in XRD suggests a polycrystalline nature of our room-temperature sputtered TiN films. Their grain size (*L*) in the (200) orientation estimated using the Scherrer’s formula^30^ regardless of substrate surface is comparable to that for the reactive sputtered TiN films reported elsewhere^19,31^: *L* = 7.33, 7.89, 9.76, and 7.89 nm for the films deposited on HfO_2_, Si, quartz, and PMMA, respectively.

**Figure 3:**
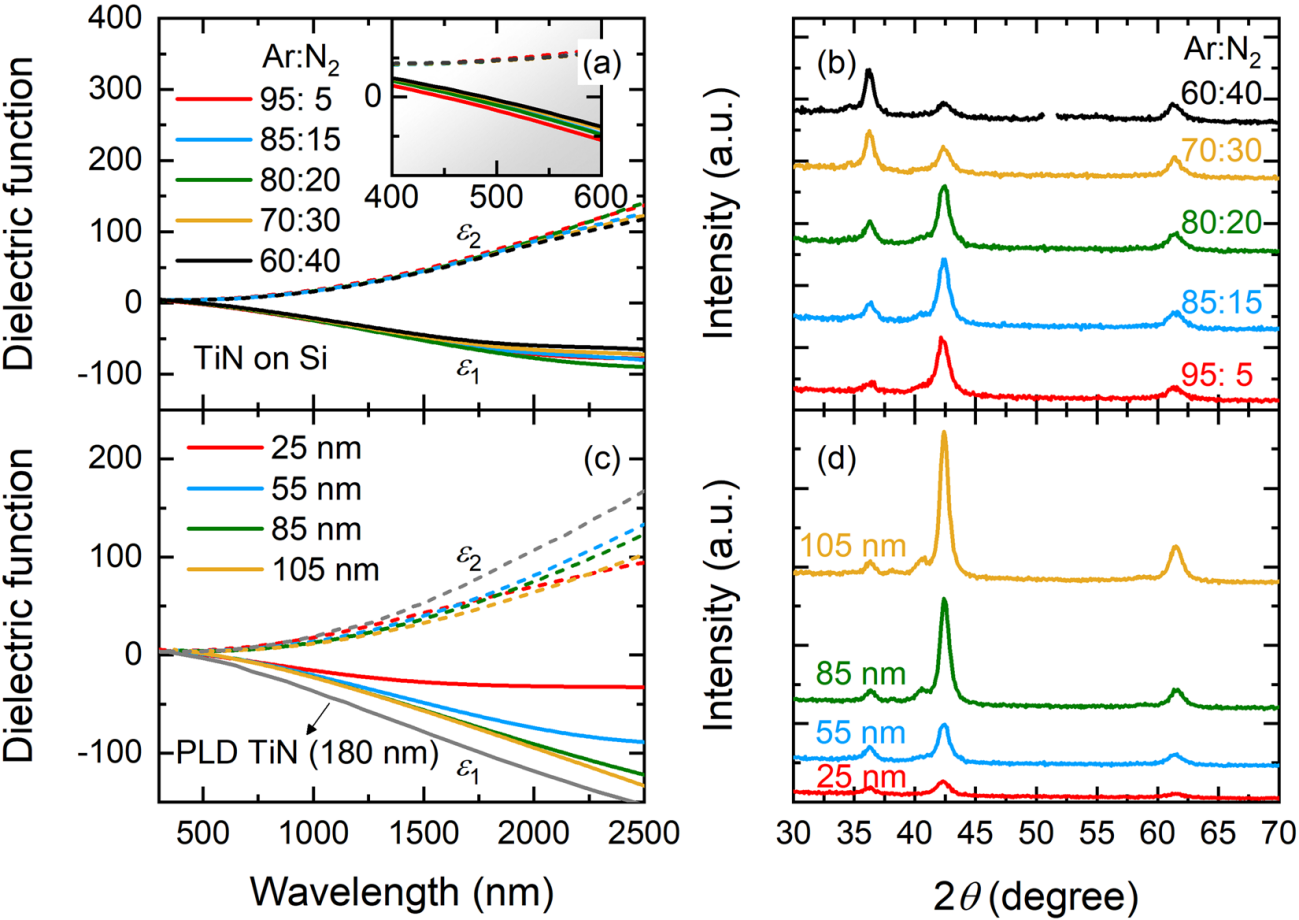
(**a**) Real *ε*_1_ and imaginary *ε*_2_ part of extracted complex dielectric functions of room-temperature sputtered TiN films on Si deposited at various Ar:N_2_ ratios. Inset: Expanded view of *ε*_1_ and *ε*_2_ at short wavelengths. (**b**) X-ray diffraction spectra of the corresponding TiN films. (**c**) *ε*_1_ and *ε*_2_ of sputtered TiN films on Si with different thicknesses. Gray solid and dashed lines: *ε*_1_ and *ε*_2_ of the TiN films by pulsed laser deposition (PLD) from ref.^39^. (**d**) X-ray diffraction spectra of the corresponding TiN films in (**c**).

In Fig. 3(a) we show the *ε*_1_ (solid lines) and *ε*_2_ (dashed lines) of the TiN films (thickness ~50 nm) deposited on Si at various Ar:N_2_ gas flow ratios (95%:5%, 85%:15%, 80%:20%, 70%:30%, and 60%:40%). As can be seen, the optical properties of the TiN films can be tuned by adjusting the Ar:N_2_ ratio in the sputtering process. The value of *ε*_1_ becomes more negative for the films deposited at higher N_2_ flow and is most negative when Ar:N_2_ reaches 80%:20%, but further increasing the N_2_ content in the reactive gas instead makes TiN films less plasmonic, essentially similar to the trend observed in the earlier work^2^. These findings are also in good agreement with high-temperature sputtered TiN films on Si and magnesium oxide (MgO) substrate, but are different from those on sapphire as well as TiN prepared in a room-temperature sputtering with a substrate bias voltage^20^. It could therefore be inferred that in addition to the Ar:N_2_ ratio, the plasmonic character of sputtered TiN films is strongly influenced by numerous other processing parameters including substrate material, deposition temperature, external biasing, and so on. In fact, we find in our experiments that the Ar:N_2_ = 80%:20% TiN films possess the most negative value of *ε*_1_ as compared to those deposited at other gas flow ratios for all substrate surfaces but quartz, for which the plasmonic character of the TiN films is optimized when Ar:N_2_ is 95%:5%. We also find that the *λ*_ps_ of our TiN films (see the inset to Fig. 3(a)) redshifts from 450 nm for the Ar:N_2_ = 95%:5% films, to 470 and 490 nm for the Ar:N_2_ = 80%:20% and the Ar:N_2_ = 60%:40% films, respectively. The observed redshift of *λ*_ps_ suggests that increasing the N_2_ flow leads to stronger dielectric screening of the free carriers in Ti 3*d* bands resulting from the interband transitions from N 2*p* to Ti 3*d* bands^32^. Moreover, the *λ*_ps_ value has been shown to be a reliable measure of the stoichiometry of TiN: *λ*_ps_ is ~468 nm (or screened plasma energy *E*_ps_ is ~2.65 eV) when the ratio of N and Ti in TiN films is near unity^33^. This suggests that our Ar:N_2_ = 80%:20% films are most likely to be stoichiometric, although others should still have close stoichiometry due to their comparable *λ*_ps_ values. In Fig. 3(b) we compare the corresponding XRD spectra of these TiN films, revealing that the Ar:N_2_ gas flow ratio has an impact on their crystalline structure. The Ar:N_2_ = 95%:5% films exhibit a predominant peak for the (200) orientation along with two small peaks for (111) and (220) orientations. The (111) peak intensity gradually increases with increased N_2_ flow, although both Ar:N_2_ = 85%:15% and Ar:N_2_ = 80%:20% films still show a (200) preferred orientation. Further increasing the N_2_ flow dramatically changes the crystalline structure of TiN, making the Ar:N_2_ = 70%:30% and Ar:N_2_ = 60%:40% films preferentially grown in the (111) orientation. These observations seem to suggest that our TiN films in the (200) preferred orientation possess better plasmonic character than (111) oriented films; however, other factors such as oxygen impurity content^20^ also need to be considered to fully elucidate the relationship between their structural properties and the corresponding plasmonic response.

We then investigate the thickness dependence of the dielectric function and crystalline structure of the TiN films deposited on Si at Ar:N_2_ = 80%:20%, as shown in Fig. 3(c,d), respectively. All TiN films show desirable plasmonic character which appears to be enhanced for thicker films, and they all exhibit clear crystalline order in the (200) preferred orientation. We do not observe any growth defects nor film cracks induced by the strain relaxation due to the lattice mismatch between TiN and Si when increasing the film thickness in our experiments. Recent studies^34,35^ have shown that the thickness dependent optical properties of plasmonic thin films arise from changes in surface-to-volume ratio, grain boundary, and carrier scattering from substrate surface as thickness varies. In particular, when films are within the ultrathin regime (thickness ≤10 nm), their plasmonic character significantly degrades due to island formation^36^, low mobility interface layers^37^ or quantum confinement effects^38^, and special thin film preparation methods such as high-temperature epitaxial growth as well as substrate treatment are thus required. However, in most of the practical plasmonic structures and devices, the thicknesses of their constituent plasmonic layers are within the thickness range of the present study (i.e., thickness ~20–100 nm). Our room-temperature sputtered TiN films can therefore be employed for a wide variety of practical applications for plasmonics due to their high plasmonic character. It is also worth noting that Sugavaneshwar *et al*.^39^ recently claimed the realization of the TiN films with the best plasmonic character reported to date by pulsed laser deposition (PLD). Despite the demonstrated high plasmonic character (see the gray solid and dashed lines in Fig. 3(c) for *ε*_1_ and *ε*_2_, respectively), their TiN films are in fact much thicker (thickness = 180 nm) than those reported in literatures. The plasmonic quality of their thinner films (thickness ~30–50 nm) remains to be verified to allow for a fair comparison.

**Figure 4:**
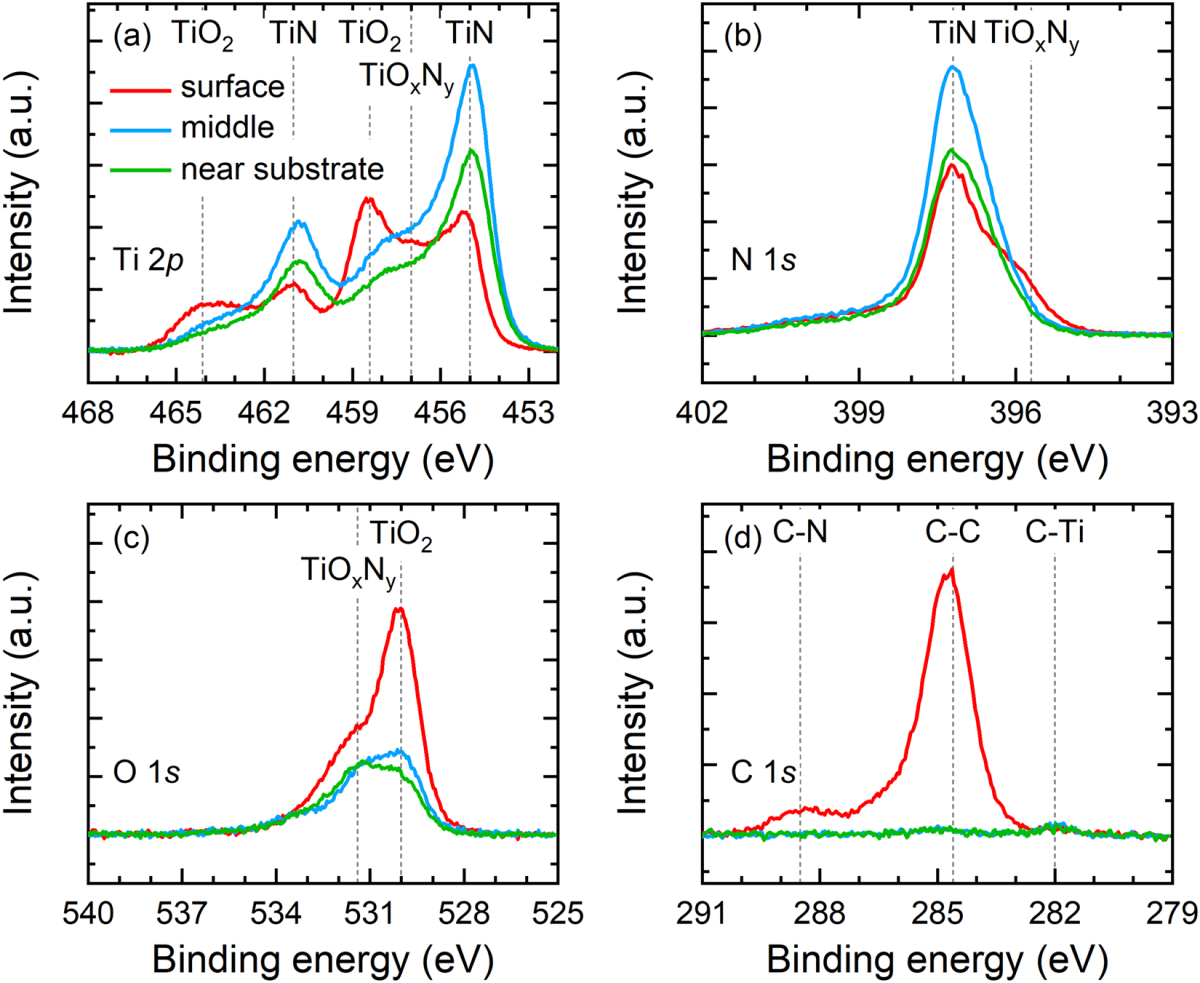
(**a**) Ti 2*p*, (**b**) N 1 *s*, (**c**) O 1 *s*, and (**d**) C 1 *s* XPS spectra of Ar:N_2_ = 80%:20% TiN films on Si substrate measured at three different depths: at the film surface (red solid curves), in the middle of the film (blue solid curves), and near the substrate (green solid curves). Vertical gray dashed lines in (**a**–**d**) mark the characteristic binding energies of different chemical states of these elements^40^.

To verify the chemical compositions of our room-temperature sputtered TiN films, we perform x-ray photoelectron spectroscopy (XPS) measurements on the Ar:N_2_ = 80%:20% films deposited on Si substrate at three different depths by *in situ* Ar ion milling: at the film surface, in the middle of the film, and near the substrate. The measured XPS spectra for Ti 2*p*, N 1 *s*, O 1 *s*, and C 1 *s* are plotted in Fig. 4(a–d), respectively. At the film surface, the Ti 2*p* doublet lines (i.e., 2*p*_3/2_ and 2*p*_1/2_) at the binding energies of ~455 and ~461 eV along with the nitrogen peak at 397.2 eV confirm the characteristic TiN phase^40^. The surface oxidation of the TiN films results in the formation of titanium dioxide (TiO_2_), manifesting as the other two peaks at 458.4 and 464.1 eV in the Ti 2*p* spectrum as well as the oxygen peak at 530 eV. The expected carbon signal detected at 284.6 eV accompanied by a small peak at 288.5 eV originates from the carbon contamination of the surface of the TiN films, as they are fully exposed to the atmosphere after growth. Moreover, the filling of the valley between the Ti 2*p*_3/2_ and 2*p*_1/2_ transitions near the binding energy of 457 eV indicates the existence of an intermediate chemical state between TiO_2_ and TiN, i.e., TiO_x_N_y_, which also appears as the shoulder at 395.7 and 531.4 eV in the N 1 *s* and O 1 *s* spectra, respectively. After etching halfway through the films, the characteristics of the TiN phase become clearly dominant while the features of the surface oxidation and carbon contamination are reduced, including the significant enhancement of the Ti 2*p* signals as well as the N 1 *s* peak for TiN, the strong suppression of both the TiO_2_ peaks in the Ti 2*p* spectrum and the oxygen peak in the O 1 *s* spectrum, and the complete elimination of the two carbon peaks in the C 1 *s* spectrum. The signals representing the TiO_x_N_y_ state are also much weaker, suggesting that the titanium oxynitride forms mostly near the film surface. By comparing the measured Ti 2*p* spectra including the spectral line shape and peak positions to those reported in the previous XP spectroscopic studies of reactive sputtered TiN films^41^, we find that the bulk of our Ar:N_2_ = 80%:20% TiN films is likely to possess high purity with a small amount of oxygen impurity. This is evidenced by the elemental compositions of the films (see Table 1) extracted from the XPS spectra: the oxygen content is ~10% in the bulk despite a higher concentration of oxygen (~26%) probed at the surface. The XPS spectra near the substrate reproduce all of the important characteristics observed in the bulk with expected reduced intensity and a strong Si signal, indicating uniform chemical compositions of the TiN films and a robust sputtering deposition process. These XPS measurement results further confirm that our room-temperature sputtered TiN differs strongly from TiO_x_N_y_ (even for the TiO_x_N_y_ films deposited at high temperature after careful chamber preparation)^25^, showing much stronger TiN characteristic signals and much less distinct features for TiO_x_N_y_.

**Table 1:** Elemental compositions of Ar:N_2_ = 80%:20% TiN films on Si substrate obtained from XPS spectra.

	Atomic percentage (%)					
	Ti 2*p*	N 1*s*	O 1*s*	C 1*s*	Si 2*p*	Ar 2*p*
surface	21.8	24.1	26.3	27.8	0.0	0.0
middle	43.8	44.0	10.2	0.0	0.0	2.0
near substrate	27.1	27.9	7.9	0.0	34.4	2.7

**Figure 5:**
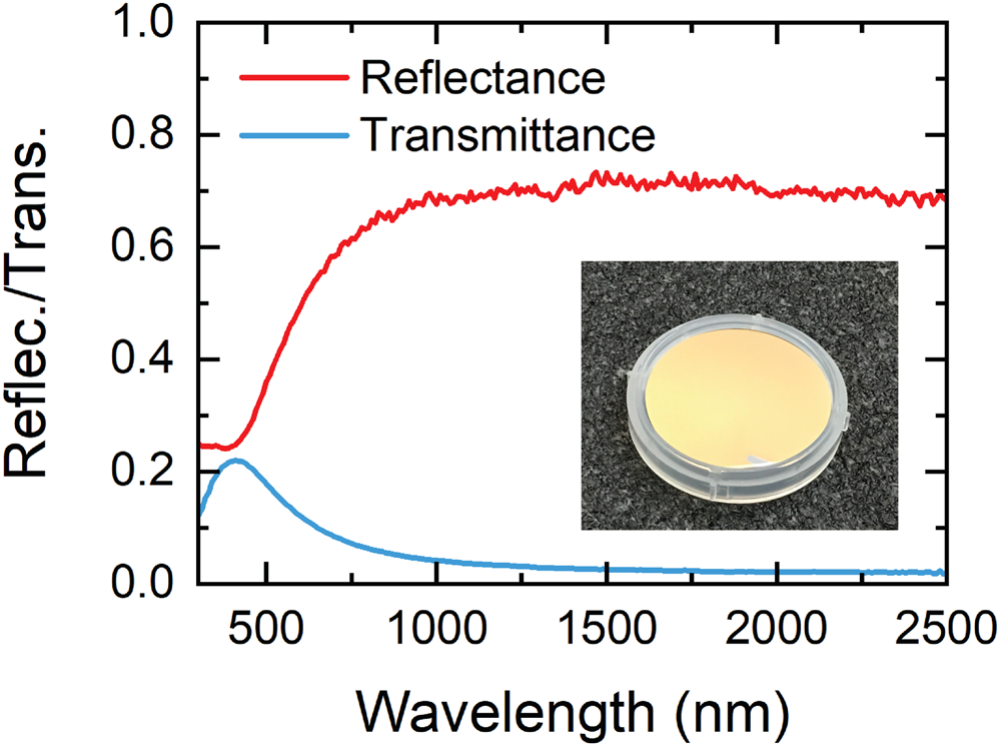
Reflectance and transmittance spectra of sputtered TiN films on quartz substrate. Inset: A photograph of sputtered TiN films exhibiting Au-like luster.

In Fig. 5 we show the unpolarized reflectance and transmittance spectra of 45 nm-thick TiN films deposited on quartz substrate at Ar:N_2_ = 95%:5%, obtained by averaging the measured p- and s-polarized reflectance/transmittance. The reflectance *R* first exhibits a minimum (*R* = 24%) at *λ* ~400 nm, and then significantly increases to *R* ≥ 70% as moving towards near-infrared wavelengths. The corresponding transmittance *T* instead reaches its maximum (*T* = 22%) at *λ* ~400 nm, and then decreases with increasing wavelength and remains <5% for *λ* > 900 nm. The observed characteristics of both reflectance and transmittance closely resemble those for Au films, validating that our TiN films are highly plasmonic/metallic. The reflectance minimum (or transmittance maximum) of TiN occurs on the high energy side where strong interband transitions prevail, whereas reflectance maximum (or transmittance minimum) in the longer wavelength region results from the high metallicity of TiN^1^. The high plasmonic quality of our TiN films is further confirmed by their Au-like luster^42^, as evident in the photograph inset to the Fig. 5.

**Figure 6:**
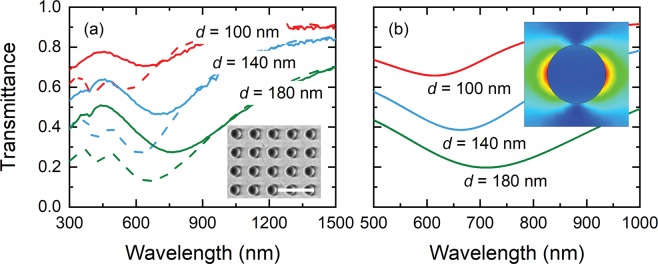
(**a**) Measured transmittance spectra of TiN (solid curves) and Au (dashed curves) nanodisk arrays with various *d*. Inset: An SEM image of TiN nanodisk arrays. Scale bar is 500 nm. (**b**) Simulated transmittance spectra of the corresponding TiN nanodisk arrays. Inset: Simulated electric field intensity of the TiN nanodisk arrays with *d* = 100 nm at the plasmonic resonance.

We fabricate two-dimensional periodic square arrays of TiN nanodisks using our room-temperature sputtered TiN films (Ar:N_2_ = 95%:5%; thickness = 45 nm) on quartz substrate. The nanodisk diameter *d* is varied from 100 to 180 nm while the period is fixed to *p* = 250 nm. In Fig. 6(a) the unpolarized transmittance spectra of the TiN nanodisk arrays with *d* = 100, 140, and 180 nm are shown (solid curves), obtained by averaging the measured p- and s-polarized transmittance. A pronounced transmittance dip is observed for all *d* values, corresponding to a plasmonic resonance mode excited in the TiN nanodisk arrays. For *d* = 100 nm, the transmittance dip occurs at *λ* = 630 nm with *T* = 71%. When *d* is larger, the observed transmittance dip clearly redshifts: for *d* = 140 nm, the transmittance dip occurs at *λ* = 690 nm with *T* = 46%; for *d* = 180 nm, the transmittance dip occurs at *λ* = 760 nm with *T* = 27%. The redshift suggests that the excited resonance is a localized surface plasmon resonance (LSPR) mode. We perform full-wave numerical simulations for the transmittance of TiN nanodisk arrays using CST Microwave Studio to gain insight into our experimental results. The simulations are carried out in frequency domain with periodic boundary conditions using the dielectric functions of TiN extracted from variable angle spectroscopic ellipsometry (VASE) measurements and online optical data^43^ for quartz. The simulated transmittance spectra are shown in Fig. 6(b), revealing overall good agreement with the experimental results. For *d* = 100 nm, the transmittance dip occurs at *λ* = 615 nm with *T* = 66%. Increasing the diameter *d* to 140 and 180 nm shifts the dip to *λ* = 663 and 711 nm and reduces the corresponding transmittance to *T* = 39% and 20%, respectively. Note that all of these resonance dips appear to be slightly stronger (i.e., lower transmittance) and occur at shorter wavelengths as compared to those observed in the experiments. This discrepancy can likely be reduced by minimizing the fabrication imperfections (see inset SEM to Fig. 6(a)) and by incorporating a thin surface oxide layer (TiO_2_ or TiO_x_N_y_) in the structural model for the fitting of the ellipsometry data of TiN. The simulated electric field intensity at the plasmonic resonance for arrays with *d* = 100 nm is shown in the inset to Fig. 6(b), indicating that the resonance is indeed an LSPR mode. Both experiments and numerical simulations therefore demonstrate that TiN based plasmonic nanostructures can be successfully realized by our room-temperature, bias-free reactive sputtering process. We further compare experimentally the plasmonic properties of the fabricated TiN nanodisk arrays to those of Au nanodisk arrays of identical geometric parameters fabricated on quartz. It is evident that the resonance dips for the fabricated TiN nanodisk arrays are similar to those for the fabricated Au nanodisk arrays (dashed curves in Fig. 6(a)) but cover a wider spectral range, proving that our TiN not only has comparable plasmonic performance to that of Au, but is more favorable for applications in the visible and near-infrared region where a broad plasmonic response is required^11^.

## Conclusions

In conclusion, we have demonstrated highly plasmonic TiN thin films and nanostructures by a room-temperature, low-power and bias-free reactive sputtering process. We investigate the optical and structural properties of our TiN films and their dependence on a number of processing parameters including substrate material, reactive gas flow ratio, and film thickness. We find that our TiN possesses one of the largest negative values of *ε*_1_ as compared to all other plasmonic TiN films reported to date. We further fabricate periodic square arrays of TiN nanodisks using room-temperature sputtered TiN films, and validate that strong localized plasmonic resonances are supported in the arrays. Our room-temperature deposition process provides an easier and more cost-effective route to realizing plasmonic TiN than other high-temperature growth methods, allows for the fabrication of more complex TiN-based plasmonic nanostructures and devices, and could potentially be integrated with the existing CMOS process technologies to enable more practical applications of plasmonics.

## Methods

Sputtering of TiN thin films was carried out at room temperature in a radio-frequency (RF) reactive sputtering system (Kurt J. Lesker) using three-inch titanium targets (99.995%) on different substrate surfaces including Si, quartz, HfO_2_, and PMMA, where HfO_2_ and PMMA thin films were first deposited on Si substrate by ALD and spin-coating, respectively. All substrates were thoroughly cleaned using either buffered oxide etchants or solvents to remove surface oxide or contaminants before loading into the chamber. The substrate-to-target distance was kept at 5 cm to ensure a high sputtering yield and an enhanced density of reactive nitrogen ions near the substrate surface. The system was then pumped down to a base pressure of 2 × 10^−7^ Torr. The Ar:N_2_ gas flow ratio was varied (Ar:N_2_ from 95%:5% to 60%:40%), while the pressure and RF power were held constant at 3 mT and 275 W respectively for all TiN films. Note that we found increasing the N_2_ content would reduce the film deposition rate, and the plasma in the sputtering process could not be sustained when further increasing the nitrogen content in the gas flow ratio to Ar:N_2_ = 40%:60%. It should also be noted that, although no intentional substrate heating was introduced, the substrate temperature during the sputtering process may have been slightly higher than room temperature, due mainly to the energy transfer from the sputtered species such as ions and neutrals from the target and the electrons from the plasma discharge. However, we expect this temperature increase should be insignificant, and the substrate temperature was much lower than that used in the low-temperature ALD work^16^. The dielectric function, thickness, transmittance (normal incidence), and reflectance (incident angle 20°) of the sputtered TiN films were measured using VASE at wavelengths from 300 to 2500 nm. XRD, XPS (VGS Thermo K-Alpha), and AFM (Bruker Innova) measurements were conducted to characterize the crystalline structure, chemical composition, and surface roughness of the TiN films, respectively. Arrays of TiN nanodisks (array area = 1 mm^2^) on quartz substrate were fabricated by electron-beam (e-beam) lithography and inductively coupled plasma (ICP) etching. Au nanodisk arrays of the same surface area were fabricated on quartz by e-beam lithography, metal evaporation, and liftoff. All of the fabricated nanodisk arrays were examined using high-resolution scanning electron microscopy (SEM), and their transmittance was measured at normal incidence using VASE.
